# Single‐Cell RNA‐Sequencing Leading to Breakthroughs in Musculoskeletal Research

**DOI:** 10.1002/jbm4.10652

**Published:** 2022-06-06

**Authors:** Noriaki Ono, Hanna Taipaleenmäki, Deborah J. Veis

**Affiliations:** ^1^ University of Texas Health Science Center at Houston Houston TX USA; ^2^ Institute of Musculoskeletal Medicine, Musculoskeletal University Center Munich (MUM), University Hospital, LMU Munich Munich Germany; ^3^ Division of Bone and Mineral Diseases, Departments of Medicine and Pathology & Immunology Washington University School of Medicine St. Louis MO USA

**Fig. 1:**
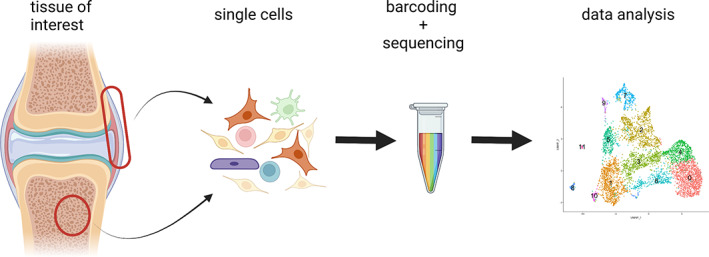
Workflow for single‐cell RNA‐sequencing analysis. Single cells are dissociated from musculoskeletal tissues of interest, barcoded in a microfluidic chip (such as 10X Chromium Single Cell Gene Expression) and sequenced by a next‐generation sequencer (such as Illumina NovaSeq 6000). The single‐cell RNA‐sequencing data are processed and analyzed by computational packages (such as Seurat).

Single‐cell RNA‐sequencing (scRNA‐seq) is a powerful approach to unravel gene expression profiles of individual cells and has become an indispensable tool in understanding cells participating in physiological and pathological processes. This approach can discover new cell populations and genes functioning within a specific group of cells at a single‐cell level. Bones and other musculoskeletal tissues are no exception. An important consideration is that currently commercialized platforms require dissociated single cells in suspension to be loaded onto droplet‐generating microfluidic devices (Fig 1).

Given the technical challenge to isolate cells from the bone at a single‐cell level, the application of this powerful technology is still at an early phase in bone research field. One of the most tractable bone‐residing cell populations is the monocyte–macrophage lineage, which are of hematopoietic origin and precursors for bone‐resorbing osteoclasts.

In this issue of *JBMR Plus*, two original articles highlight the power of scRNA‐seq approaches in discovering new macrophage subtypes and identifying new genes functioning in osteoclast precursor cells of both humans and mice. In the first article by Fujii and colleagues,^(^ ^1^ ^)^ the authors identified a novel “surgery‐induced” highly inflammatory CD9^+^ IL1^+^ macrophage population in a mouse model of anterior cruciate ligament reconstruction surgery, which resolved early and became replaced by another type of macrophages. In the second article by Omata and colleagues,^(^ ^2^ ^)^ the authors identified a GTPase family member, RAB38, as a highly expressed molecule in both human and murine osteoclast precursor cells. This protein was associated with dynamic changes in histone modification and transcriptional regulation. Fujii and colleagues evaluated bone healing of mice lacking *Ccr2*, which they identified as one of the markers for late‐responding macrophages after the surgery. Omata and colleagues silenced *Rab38* expression using short hairpin RNA and evaluated osteoclast differentiation in vitro. These initial validation studies placed the computational findings in a biological context, although much remains to be done to fully understand the implications of the large data sets.

We appreciate that functional validation is a very challenging step, and comprehensive characterization of identified cells or proteins may take many months or even years. As a fully open‐access journal dedicated to rapid dissemination of important findings in musculoskeletal research, *JBMR Plus* understands the need to publish exciting discoveries quickly. Rapid publication allows the entire research community to think about the data and use it in a variety of ways, advancing the field as a whole. Therefore, we welcome technically rigorous studies that describe large data sets, albeit with somewhat limited functional validation. The two articles in this issue describing scRNA‐seq data sets are great examples of the type of studies that *JBMR Plus* seeks to publish. Analogous studies employing analyses such as proteomics and metabolomics generating equally impactful data sets will also be welcomed. We hope that investigators in the musculoskeletal research community will utilize *JBMR Plus* to disseminate and discover important work in a timely manner.
